# “Feeling the Blues”: A Case of Calcium Channel Blocker Overdose Managed With Methylene Blue

**DOI:** 10.7759/cureus.19114

**Published:** 2021-10-29

**Authors:** James R Pellegrini, Rezwan Munshi, Muhammad S Tiwana, Tinu Abraham, Hira Tahir, Najia Sayedy, Javed Iqbal

**Affiliations:** 1 Internal Medicine, Nassau University Medical Center, East Meadow, USA; 2 Pulmonary and Critical Care, Nassau University Medical Center, East Meadow, USA

**Keywords:** shock, insulin, methylene blue, overdose, amlodipine

## Abstract

Amlodipine is a dihydropyridine calcium channel blocker (CCB) commonly used to treat hypertension. In the United States, approximately 9,500 cases of CCB intoxication due to deliberate or inadvertent overdose were reported to poison centers in 2002. We present a case of a patient who presented with CCB overdose complicated by acute respiratory distress syndrome (ARDS) and recalcitrant shock all of which resolved with methylene blue therapy. We present a case of a 56-year-old African American woman who presented to the emergency department (ED) after intentional ingestion of large amounts of multiple pills likely consisting of cyclobenzaprine, amlodipine, losartan, and ibuprofen following an argument with her boyfriend. Treatment included insulin drip, 10% dextrose, and norepinephrine drip which was titrated up. First insulin drip and 10% dextrose were titrated up; however, vasopressor-resistant hypotension persisted, and the decision was made to administer methylene blue. Over 9,500 cases of CCB toxicity were reported to poison centers in the US in 2002. Although no definitive treatment is outlined, first-line therapy consists of IV calcium, high-dose insulin, and vasopressor support with either norepinephrine or epinephrine. Traditionally, methylene blue is used for methemoglobinemia and in cardiothoracic ICUs for post coronary artery bypass vasoplegia. It acts by selectively inhibiting nitric oxide-activated cyclic guanylate cyclase leading to decreased vasodilation of arteriolar smooth muscles improving vascular tone and systemic vascular resistance. In severe amlodipine overdose, experimental models demonstrate methylene blue improves HR and mean arterial pressure (MAP), improving survival rate. With few adverse side effects (green-tinged discoloration of urine, saliva, tears, and bodily fluids), methylene blue should be explored and implemented in the treatment of CCB overdose with refractory hypotension and ARDS.

## Introduction

For more than two decades, amlodipine has helped to treat elevated blood pressure and angina. Amlodipine is a dihydropyridine calcium channel blocker (CCB), inhibits the movement of calcium ions through vascular smooth muscle cells and cardiac muscle cells which in turn prevents contraction of these cells [1]. Adverse effects of CCB include vasodilatory effects such as peripheral edema, dizziness, palpitation, and flushing. Along with beta-blockers and digoxin, CCBs are one of the most common drugs people use to overdose. Although there is no definite guideline for the management of CCB overdose, extrapolated treatment includes activated charcoal, IV fluids, insulin, glucagon, and vasopressors [2]. Specifically, high doses of insulin along with glucose have shown to be useful and are the suggested first-line treatment for CCB overdose [2]. We present a case of a patient who presented with CCB overdose complicated by acute respiratory distress syndrome (ARDS) and recalcitrant shock all of which resolved with methylene blue therapy.

## Case presentation

**Figure 1:**
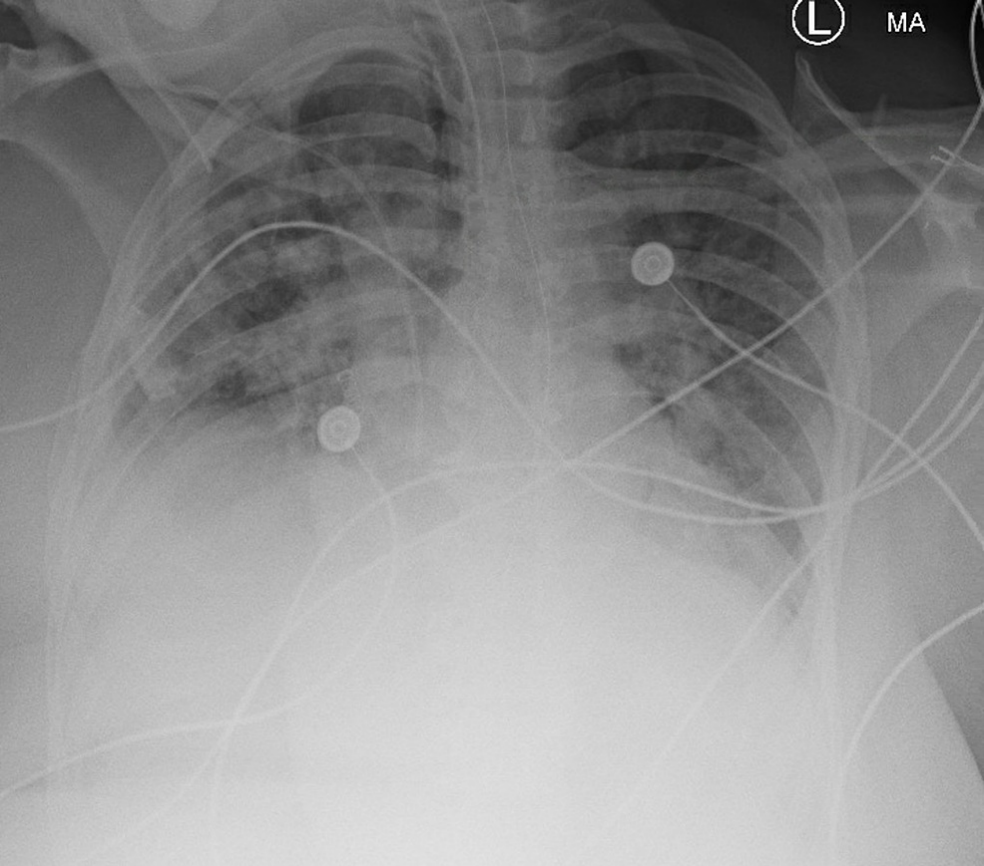
Chest x-ray depicting acute respiratory distress syndrome

Our patient is a 56-year-old African American female with a past medical history of hypertension and transient ischemic attack who presented to the emergency department (ED) after intentional ingestion of large amounts of prescription medications. History obtained from ED physicians and emergency medical services (EMS) revealed the patient had an argument with her boyfriend and, in a suicidal attempt, ingested multiple pills most likely consisting of cyclobenzaprine, amlodipine, losartan, and ibuprofen. Prior to EMS arrival, the police administered 4 mg intranasal Narcan without any improvement of neurologic status. The patient was found obtunded, non-responsive to painful stimulation, hypotensive during transit to hospital by EMS. Upon ED arrival, the patient’s Glasgow Coma Scale score was 7, and vitals were as follows: blood pressure was 60/40 mmHg, pulse was 64, respiratory rate was 10, saturating 90% oxygen on room air. The patient was subsequently intubated for airway protection, a central line was placed along with the initiation of vasopressors. The patient received 2 g of calcium chloride and 2 mg of glucagon and was subsequently transferred to the Medical Intensive Care Unit (MICU). Treatment included Insulin drip running at 100 units/hour (U/hr), 10% dextrose solution running at 100 cc/hr, as well as norepinephrine drip titrated to maintain a mean arterial pressure (MAP) > 65 mmHg. Throughout the evening, the patient required an increasing amount of norepinephrine. Instead of adding a second vasopressor, the insulin drip was increased to 200 U/hr along with increasing 10% dextrose to 200 cc/hr. The following day, the patient had vasopressor-resistant hypotension and a decision was made to administer Methylene blue 140 mg intravenous push once. Several hours later the patient's vital signs began to improve and vasopressors and insulin drip were slowly titrated down. However, her hospital course was further complicated by worsening hypoxia with chest radiographs demonstrating new, bilateral patchy airspace opacities consistent with ARDS (Figure 1).

By day 7 of hospitalization, the patient's hemodynamic and respiratory status significantly improved, she was weaned off vasopressors and successfully extubated and transferred to Medicine floors for further management. After evaluation and clearance by Psychiatry, the patient was discharged from the hospital and provided with appropriate follow-up appointments.

## Discussion

CCBs are used in the treatment of hypertension, angina pectoris, cardiac arrhythmias, and other disorders. These medications are available in both immediate-release and extended-release preparations. Conventionally used CCBs belong to three main chemical classes, with each subclass having differing affinities for cardiac tissue and vascular smooth muscle; phenylalkylamines (i.e., verapamil), benzothiazepines (i.e., diltiazem), and dihydropyridines (i.e., amlodipine). 

The potential toxicity of these agents is significant, and the general population is typically unaware of it. In the United States, for example, approximately 9,500 cases of CCB intoxication due to deliberate or inadvertent overdose were reported to poison centers in 2002 [1,2]. CCBs were responsible for just 16% of all cardiovascular medication toxicities, with 38% of those resulting in fatalities [3].

L-type voltage-gated calcium channels are targeted by CCBs in all of their subtypes. Because they are lipophilic, bind quickly to plasma proteins, and undergo substantial hepatic first-pass metabolism, CCBs are very effectively absorbed orally. CCBs have a wide distribution volume, and removal by hemodialysis or hemofiltration is frequently unsuccessful [4,5]. As hepatic clearance shifts from first-order to zero-order kinetics at larger dosages, clearance slows [4,5].

All subclasses of CCBs suppress pancreatic insulin production and cause end-organ insulin resistance, resulting in hyperglycemia. This hyperglycemia can be used as a clinical indicator of the degree of poisoning [6,7]. CCBs also inhibit calcium-stimulated mitochondrial activity and glucose catabolism, resulting in lactate formation and ATP hydrolysis, which contributes to metabolic acidosis [8]. Hypotension and bradycardia, when progressive, can eventually lead to cardiogenic shock [9]. Mild hypokalemia and mild to severe hypocalcemia are other frequent complications. Seizures, myocardial infarction, ARDS, renal failure, intestinal infarction, and stroke can all occur as a result of significant hypoperfusion and end-organ ischemia caused by a severe overdose [10].

CCB poisoning can be treated in a variety of ways: Reduced gastrointestinal absorption has been found to be beneficial when activated charcoal in a dosage of 1 g/kg is given within one to two hours. Charcoal treatment two hours after amlodipine consumption decreased absorption by 49% relative to controls in a volunteer trial [11,12]. Calcium administration is justified by the fact that increasing extracellular calcium concentration promotes calcium influx through unblocked L-type calcium channels. With severe poisoning, however, responses are varied and poor [13-15].

G proteins activate adenylate cyclase, resulting in a positive chronotropic and inotropic impact from glucagon produced by pancreatic alpha cells [9,10]. In animal models of CCB overdose with glucagon, there was an improvement in heart rate, cardiac output, and reversal of AV blocks [16]. The effects of administration become apparent in one to three minutes and continue for 10 to 15 minutes [16].

Hyperinsulinemic euglycemia (HIE) has proven to be an effective treatment for severe CCB poisoning. Experimental models show that CCB toxicity causes myocardial substrate preference to shift from free fatty acids to carbohydrates, resulting in decreased cardiac substrate delivery [17,18]. CCBs also inhibit glucose metabolism, causing lactic acidosis and metabolic acidosis by reducing insulin production, creating tissue insulin resistance, and interfering with glucose metabolism. Insulin given in this context aids in the reversal of all of these metabolic abnormalities while also having a direct beneficial inotropic impact [17,18]. Blood glucose and potassium levels should be monitored before starting insulin therapy, and if they are less than 200 mg/dL and 2.5 meq/L, respectively, dextrose and potassium supplementation are warranted. 1 U/kg regular insulin intravenous bolus followed by 1 to 10 U/kg/hr continuous infusion is the current insulin dosage guideline [19-22]. The objective of treatment is to establish hemodynamic stability and vasoactive agent withdrawal.

Loss of peripheral vascular resistance and cardiac depression accompany severe CCB poisoning, resulting in refractory hypotension and shock. In addition to the various pharmaceutical treatments, catecholamine infusion may be required in this situation. Between dopamine, norepinephrine, epinephrine, and even dobutamine, there is no one recognized agent of choice [23]. As a result, selections should be based on the cause of shock and an assessment of cardiac function. In the context of vasodilatory shock, the Society of Critical Care Medicine consensus recommendations proposes administering norepinephrine or epinephrine, with norepinephrine being preferred [23,24].

Methylene blue is commonly used to treat methemoglobinemia and post-coronary artery bypass vasoplegia in cardiothoracic intensive care units (low systemic vascular resistance) [25]. It works by blocking cyclic guanylate cyclase that is triggered by nitric oxide. This reduces arteriolar smooth muscle vasodilation, enhancing vascular tone and systemic vascular resistance [25]. Experimental models show that methylene blue improves HR and MAP in severe amlodipine overdose, increasing survival rates [25,26]. As an adjunct to vasopressors and HIE treatment, it has successfully treated refractory cases of CCB overdose [25,26].

The quantity of intake, the severity of toxidrome, and the degree of organ dysfunction all influence the prognosis beyond acute toxicity [9]. Advanced age, previous impairment of cardiac function, multi-organ failure, and refractory shock necessitating salvage treatments such as extracorporeal membrane oxygenation (ECMO) are all poor prognostic factors [9]. Arrangements for proper psychiatric examination and behavioral health consulting may be appropriate once the patient has been stabilized.

## Conclusions

Hospitalizations due to lethal overdose have been increasing across the nation. It is important to recognize CCB overdose because prompt treatment improves survival in these patients. Although there is no defined method of resuscitation, there are many suggested treatment options. First-line therapy consists of IV calcium, high-dose insulin, and vasopressor support with either norepinephrine or epinephrine. The case presented demonstrates the success of methylene blue as a treatment modality for CCB overdose resulting in ARDS and recalcitrant hypotension. With only a few adverse side effects (including green-tinged discoloration of urine, saliva, tears, and bodily fluids), methylene blue should be further explored and implemented in the treatment of CCB overdose with refractory hypotension and ARDS.
